# Artificial Intelligence Based Multimodality Imaging: A New Frontier in Coronary Artery Disease Management

**DOI:** 10.3389/fcvm.2021.736223

**Published:** 2021-09-22

**Authors:** Riccardo Maragna, Carlo Maria Giacari, Marco Guglielmo, Andrea Baggiano, Laura Fusini, Andrea Igoren Guaricci, Alexia Rossi, Mark Rabbat, Gianluca Pontone

**Affiliations:** ^1^Centro Cardiologico Monzino, Istituto di Ricovero e Cura a Carattere Scientifico (IRCCS), Milan, Italy; ^2^Department of Clinical Sciences and Community Health, Cardiovascular Section, University of Milan, Milan, Italy; ^3^Department of Emergency and Organ Transplantation, Institute of Cardiovascular Disease, University Hospital Policlinico of Bari, Bari, Italy; ^4^Department of Nuclear Medicine, University Hospital Zurich, Zurich, Switzerland; ^5^Center for Molecular Cardiology, University Hospital Zurich, Zurich, Switzerland; ^6^Department of Medicine and Radiology, Division of Cardiology, Loyola University of Chicago, Chicago, IL, United States; ^7^Department of Medicine, Division of Cardiology, Edward Hines Jr. VA Hospital, Hines, IL, United States

**Keywords:** artificial intelligence, coronary artery disease, multimodality imaging, machine learning, deep learning, radiomics

## Abstract

Coronary artery disease (CAD) represents one of the most important causes of death around the world. Multimodality imaging plays a fundamental role in both diagnosis and risk stratification of acute and chronic CAD. For example, the role of Coronary Computed Tomography Angiography (CCTA) has become increasingly important to rule out CAD according to the latest guidelines. These changes and others will likely increase the request for appropriate imaging tests in the future. In this setting, artificial intelligence (AI) will play a pivotal role in echocardiography, CCTA, cardiac magnetic resonance and nuclear imaging, making multimodality imaging more efficient and reliable for clinicians, as well as more sustainable for healthcare systems. Furthermore, AI can assist clinicians in identifying early predictors of adverse outcome that human eyes cannot see in the fog of “big data.” AI algorithms applied to multimodality imaging will play a fundamental role in the management of patients with suspected or established CAD. This study aims to provide a comprehensive overview of current and future AI applications to the field of multimodality imaging of ischemic heart disease.

## Introduction

Cardiovascular disease represents one of the leading causes of morbidity and mortality in the world (1). In 2017, coronary artery disease (CAD) affected 1.72% of the global population and was recognized as the leading cause of death (1).

This highlights the need of an effective and efficient diagnostic-therapeutic path for the diagnosis and risk stratification of CAD patients.

CAD management has dramatically changed over the past few decades. Currently, invasive coronary angiography remains the gold standard for patients with a high risk of CAD allowing for both the diagnosis and potential for therapeutic intervention. However, this strategy is time consuming and prone to intra- or periprocedural risks (e.g., bleeding risk, puncture site bleeding, coronary artery dissection, radiation exposure, and contrast induced nephrotoxicity) and thus is typically not recommended as a first line strategy for patients at low-to-intermediate risk for CAD.

In this category of patients, multimodality imaging is assuming an increasingly important role, as outlined in the latest ESC guidelines on the management of chronic coronary syndromes (CCS) (2), with the aim of both improving the early detection of significant asymptomatic CAD and making the diagnostic workflow more efficient; for example, by avoiding a large number of negative invasive coronary angiograms.

With the increasing availability of powerful computers and large datasets, the implementation of artificial intelligence (AI) in the current workflow of multimodality imaging for CAD diagnosis appears a promising tool in aiding cardiologists and radiologists in the growing demand of cardiovascular imaging examinations.

In this review, we will explore the current AI applications of multimodality imaging applied to CAD management, highlighting the great potential and possible pitfalls of this new frontier in CAD management.

## Basic Concepts of Artificial Intelligence

The term AI outlines the ambitious attempt to replicate with a machine the most distinctive feature of the human being, its ability to think. In order to simulate the characteristics of human thought, an AI algorithm must be able to perform tasks considered distinctive of a human being: to understand a language, to recognize images, to identify known objects, to solve problems, and to learn from its own mistakes.

The concept of AI was first mentioned in 1956 (3). At that time its implementation in real life appeared to most people as a distant futuristic utopia. However, AI applications are rapidly entering everyday life (4–6) and the medical field, possibly representing a new frontier for the evolution of our society.

AI algorithms can be developed with the increasing availability of large amounts of data (“big-data”) and powerful computational machines.

AI applications are based on two main methods: machine learning (ML) and deep learning (DL).

ML is a technique that provides AI algorithms the ability to learn when exposed to large datasets of correctly classified features. Beyond the quality of the algorithm itself, the quality of the characterization of the data and their heterogeneity are crucial factors for the real-world application of the algorithm. For this reason, ML applications are first developed on training and validation datasets and then tested in an independent dataset to verify their adaptability for use outside the domain in which they were developed.

Two main models of ML have been developed to date: supervised and unsupervised learning. The main difference between these two methods resides in the presence or absence of a prefixed outcome. In supervised learning the AI model navigates the dataset to find the best combination of features that fits with the prefixed outcome; while in unsupervised learning the algorithm simply tries to discover any potential consistent pattern concealed in the dataset (6).

Examples of ML supervised learning methods are regression analysis, support vector machines (SVM) and random forests (RF); while unsupervised learning is funded on principal component and cluster analysis approaches. A more detailed explanation of these concepts goes beyond the scope of this review (6).

DL can be considered a particular subset of ML that uses multiple artificial neural networks to directly interrogate datasets to make predictions. In the medical imaging context, the most widely DL network is represented by Convoluted Neural Network (CNN), a network of multiple interconnected layers that roughly mimics the functioning of the visual human cortex (6). In the context of cardiovascular imaging both ML and DL have been applied. The former has mainly been used to predict diagnostic or prognostic outcomes and bases the analysis on datasets of manually labeled image features; while the latter have directly been applied to images in order to automatically obtain diagnoses (6, 7).

Early-stage AI applications were deployed to automate time-consuming medical tasks to reduce workload (e.g., to shorten image acquisition, image analysis and reporting time); more recently their development has been focused to more complex duties, such as to perform autonomous diagnoses and risk-stratification (8).

In this context, in recent years a new technique called radiomics has emerged as a new tool to combine with traditional AI applications to dig deep into the images to identify possible risk predictors or unearth features that can lead to early diagnoses. Radiomics is able to convert every voxel of a digital medical image in a high amount of quantitative mathematical imaging data that can be later analyzed by high-performance computers and AI algorithms (9, 10). This analysis can aid the human operator to see beyond the limit of its eye, revealing textures concealed behind medical images; when combined together, this information can be automatically quantified and analyzed by AI with a process called “texture analysis” that can lead to new diagnostic tools or prognostic models.

## AI Applications in the Clinical Workflow of Cad Patients

The most recent ESC guidelines on CCS base the choice of the diagnostic tool for CAD detection on the pre-test individual clinical likelihood of disease, in order to select the most appropriate invasive or non-invasive diagnostic test to perform, according to individual patient characteristics (2).

**Figure 1:**
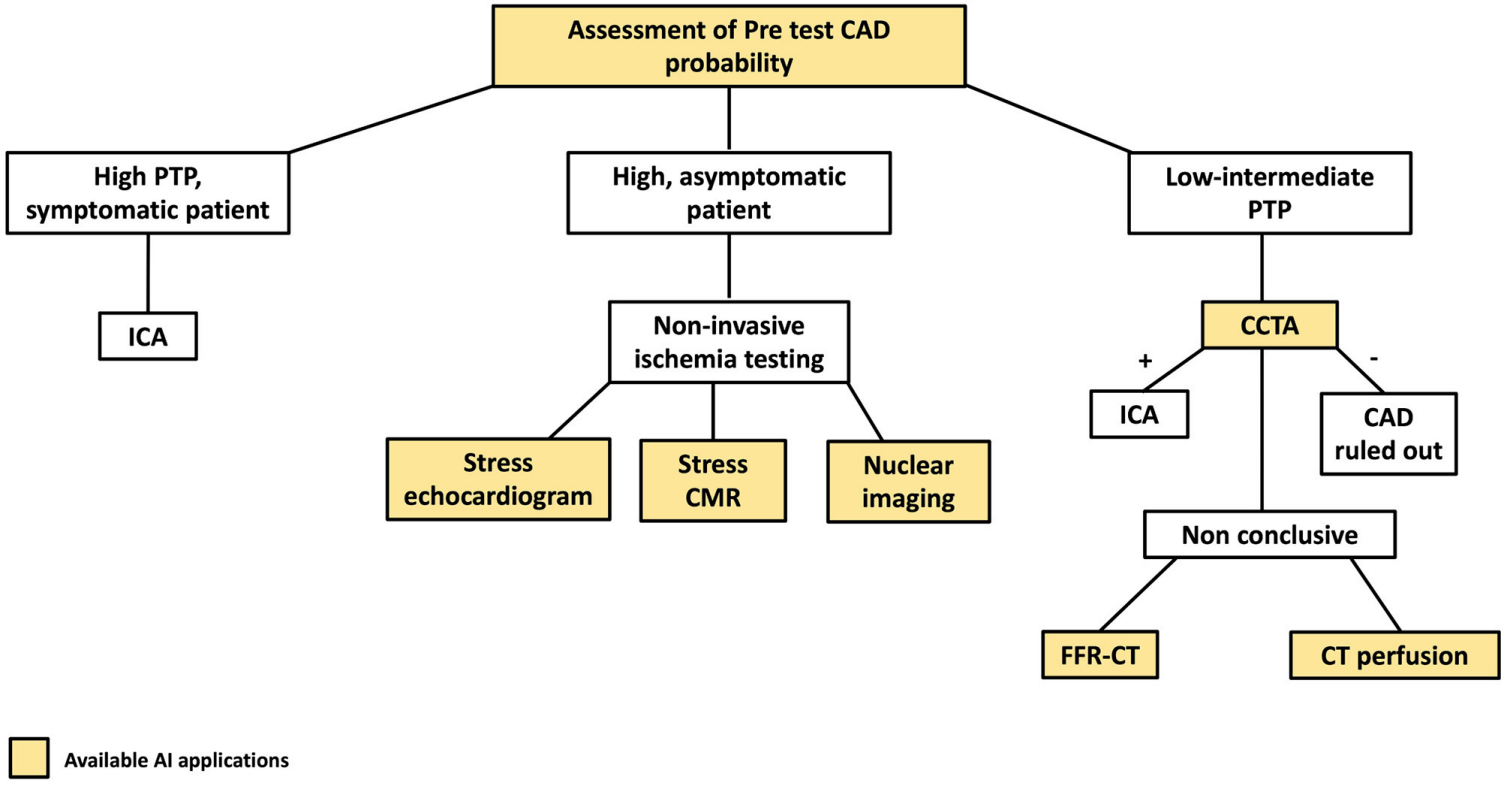
AI applications to the coronary artery disease (CAD) clinical workflow. The figure describes the stages of the clinical CAD workflow in which AI have found applications. PTP, pre-test probability; CCTA, coronary computed tomography angiography; ICA, invasive coronary angiography.

Figure 1 highlights AI applications deployed in all the steps of the diagnostic definition of CAD, from pre-test risk definition to their implementation in individual imaging methods used in clinical practice for CAD assessment.

## AI Applications in Cad Pre-Test Likelihood Definition

The last ESC Guidelines base the definition of the pre-test individual likelihood of CAD from a pooled analysis of clinical and demographic characteristics (i.e., age, sex, and the nature of symptoms) of 15,815 patients symptomatic for chest pain (2). Many clinical models that incorporate information on clinical risk factors for CVD, resting ECG changes, or coronary artery calcification have improved the identification of patients with obstructive CAD and the Guidelines recognize these factors as an integrated part of cardiovascular risk evaluation, to better identify a personalized clinical likelihood of CAD.

In addition, imaging parameters such as coronary artery calcium (CAC) score and epicardial adipose tissue (EAT) quantification are assuming a role of increasing importance in the quantification of cardiovascular risk.

In this paragraph, we will summarize the principal AI applications developed for the automatic quantification of CAC and EAT (Table 1).

**Table 1:** Main AI powered imaging methods for the definition of the pre-test likelihood of CAD.

**References**	**Summary**	**Performance**
**CAC scoring**		
Takx et al. (11)	Automated CAC scoring on non-contrast-enhanced, non-gated chest CT recorded for lung cancer screening	*k* = 0.85 for Agatston risk categories between the automated and reference scores
Wolterink et al. (12)	Automated per patient and per coronary artery CAC scoring	High ICCs (0.98 for LAD; 0.69 for LCx and 0.95 for RCA) for CAC volume scoring compared with manual scoring
Lessmann et al. (13)	Automated CAC scoring on low-dose chest CT recorded for lung cancer screening	*k* coefficient = 0.9 for risk category assignment based on per subject coronary artery calcium
Sandstedt et al. (14)	Automated CAC scoring on non-contrast CT images	High correlation (ρ = 0.935) between AI and traditional Agatston score determination
van Velzen et al. (15)	Automated CAC scoring automatically adapting to non-contrast CT scans performed with multiple acquisition protocols	ICCs of 0.79–0.97 for CAC scoring among different scan types and *k* = 0.9 in patients' risk stratification according to Agatston score
Zeleznik et al. (16)	Automated CAC scoring on CT scans performed with multiple acquisition protocols and in different clinical scenarios	High correlation (ρ = 0.92) with manually measured CAC scores; accurate risk stratification for CVE across CT scans acquired with different protocols, in patients with different clinical presentations*
**EAT analysis**		
Commandeur et al. (17)	Automated EAT quantification	High correlation (ρ = 0.97) with manual quantification
Commandeur et al. (18)	Prediction of hard CVE though a ML algorithm	Higher AUC for the AI application compared to clinical risk scores (0.82) and CAC score (0.77)
Eisenberg et al. (19)	MACE prediction through a fully automated EFV and attenuation quantification	Increased EAT volume and decreased EAT attenuation were both independently associated with MACE (HR 1.35 and 0.83, respectively)

*AUC, area under the curve; CAC, Coronary artery calcium; CVE, cardiovascular events; EAT, epicardial adipose tissue; EFV, epicardial fat volume; HR, hazard ratio; k, correlation coefficient; ICCs, intra class correlations; MACE, major cardiovascular events; ρ, Spearman correlation coefficient*.

* *In the context of both primary and secondary CAD prevention and both in patients with acute and chronic chest pain*.

### AI Applications for Coronary Artery CAC Scoring

CAC score is a well-established predictor of obstructive CAD, particularly useful in identifying patients with high CV risk, independent of clinical risk assessment scores.

CAC scoring requires dedicated software for semi-automatic image segmentation and time demanding manual measurement by trained experts on a dedicated ECG-gated cardiac CT. This time-consuming approach is not feasible for everyday clinical practice and hinders the application of CAC score on non-targeted routine chest CT, despite the demonstration of its good reliability on non-targeted CT exams (20), thus limiting the large-scale application of CAC score as a screening method for CAD.

The application of AI algorithm for CAC scoring in dedicated non-contrast-enhanced, ECG-gated CT scans is feasible, as demonstrated by Sandstedt et al. (14), who demonstrated an excellent comparability of a fully automated CAC score AI application vs. a traditional semi-automated measurement in 315 CAC-scoring dedicated CT scans (*r* = 0.935 for Agatston score assessment between the two methods). Similarly, Wolterink et al. (12), developed a ML approach that automatically quantified total patient and per coronary artery calcifications and selected the most complex cases to be reviewed by experts. This system led to an excellent intra-class correlation coefficient between the manual and the AI determined coronary artery CAC volume of 0.95. Similar results were obtained for CAC volume for each epicardial coronary artery.

CAC score analysis can express its full potential as a screening tool if applied on a large scale, even in examinations not aimed at cardiac analysis, as in the case of patients undergoing low-dose chest CT for cancer screening or follow-up.

In this context, the application of ML and DL algorithms has proven their efficacy in ensuring automatic measurement of CAC score values in large datasets of low-dose, non-ECG gated CT scans (>1,500 CT scans) performed for lung cancer screening (11, 13).

Based on the demonstration that AI applications were reliable in quantifying CAC on CT scans not targeted for that scope, Van Velzen et al. (15) demonstrated how a DL method can adapt to different types of CT examinations and acquisition protocols, if trained to do so. In this study, the authors elaborated a DL algorithm composed of two consecutive CNN. The algorithm was then trained on large datasets of more than 7,000 CT acquired with different CT protocols. The DL application showed a remarkable correlation with manual CAC scoring, both in correctly identifying CAC among different scan types (internal class correlation comprised between 0.79 and 0.97) and in correctly risk stratifying patients according to their Agatston score (*k* correlation for all test= 0.9).

Recently, a study by Zeleznik et al. (16) confirmed the possibility to broadly use AI applications to use CTs acquired in different clinical scenarios to screen for CAD using CAC score. The authors first developed a DL application trained to identify and quantify CAC based on manual segmentations performed by expert CT readers on 1,636 cardiac CT scans. Two CNN were trained for the correct localization and segmentation of the heart and then tested among CT scans acquired with different protocols. The DL application not only demonstrated high correlation with manually measured CAC scores (rho = 0.92) in a cohort of 5,521 patients, but accurately stratified the risk for cardiovascular events across a large test cohort of 19,421 patients with different clinical presentations (from primary to secondary CAD prevention and acute to chronic chest pain settings) and different CT scan acquisition protocols (predicted AUC for automated and manual CAC score event prediction were 0.74 and 0.75, respectively, *p* = 0.544).

AI powered CAC score has the potential to become a fundamental tool for risk stratification of patients with suspected acute or chronic CAD, helping the clinician in correctly defining the cardiovascular risk profile of each individual patient.

### AI Applications for Epicardial and Pericoronary Adipose Tissue Characterization

**Figure 2:**
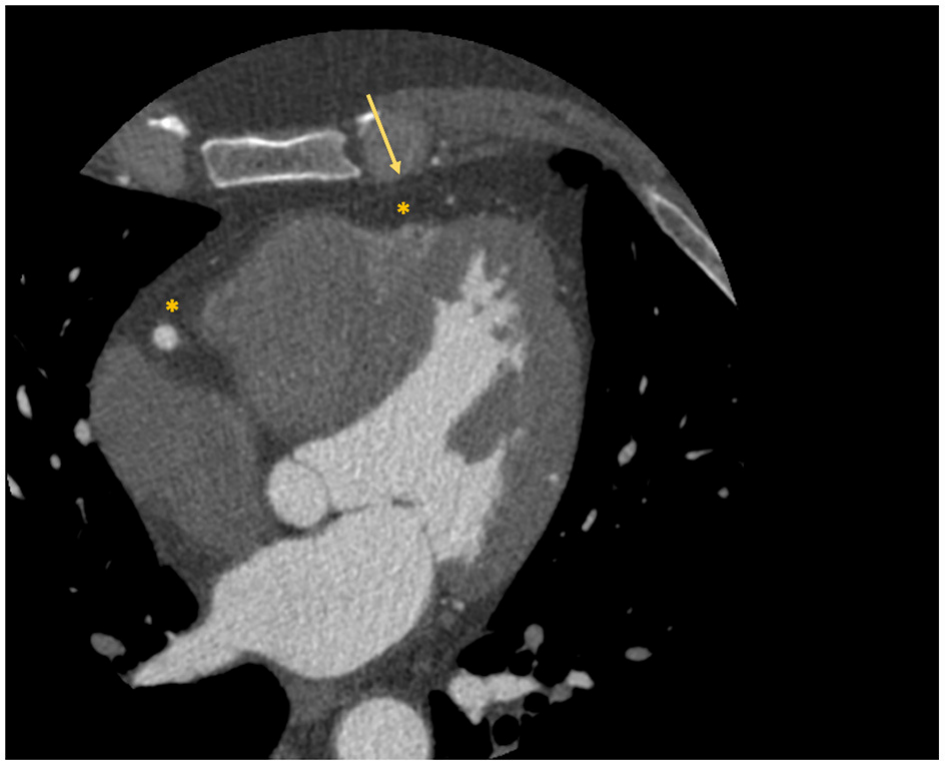
Localization of epicardial adipose tissue at cardiac CT scan. The figure depicts an example of visualization of the epicardial adipose tissue with a cardiac CT scan. The asterisk identifies the hypodense area of adipose tissue; while the arrow identifies the visceral pericardium.

Interest has grown toward the correct quantification and analysis of the epicardial adipose tissue (EAT), namely the fat layer located between the myocardium and the visceral pericardium (Figure 2), due to the emerging evidence that identified its role in atherosclerosis development and consequently in obstructive CAD (21). An even more important role in atherosclerosis seems to be played by the pericoronary adipose tissue (PCAT), the EAT layer directly surrounding the coronary arteries.

In physiological conditions it is fundamental in maintaining the homeostasis of the vascular wall; while when dysfunctional (e.g., in inflammatory conditions) it plays a key role in atherogenesis by the production of pro-inflammatory cytokines.

Various cardiac imaging modalities are capable of quantifying EAT, ranging from traditional echocardiography to CMR and cardiac computed tomography. The latter has recently become the key modality in this field due to its ability not only to visualize and precisely quantify EAT, but also to assess the coronary arteries and the PCAT simultaneously.

A paper by Antonopoulos et al. (22) demonstrated the possibility to identify this inflammatory process with CCTA *via* an imaging biomarker called “fat attenuation index (FAI).” The authors showed that PCAT signal attenuation was a biomarker of adipose tissue inflammation and also demonstrated that FAI correlated with the presence of CAD and was associated with stenosis >50%.

AI applications have been developed to automate the processes of EAT and PCAT quantification and characterization.

Since conventional EAT measurement by semi-automated software can be time-consuming, several AI applications have been developed to shorten this process (17, 23, 24), proving their ability to correctly quantify EAT from non-contrast cardiac CT scans.

In 2020, two studies demonstrated the ability of AI powered solutions to improve patients cardiovascular risk stratification with the implementation of information regarding EAT in the analysis of non-contrast cardiac CT.

Commandeur et al. (18) created a ML algorithm that integrated clinical variables, CAC score and EAT quantification to predict hard cardiovascular events (i.e., MI or CV death) during a mean follow-up of 14.5 years in a large population of 1,912 asymptomatic subjects from the EISNER trial. The AI algorithm clearly outperformed both well-established clinical risk scores and CAC score in CV event prediction.

Similarly, Eisenberg et al. (19) confirmed the predictive value of the DL assessment of EAT volume as an independent cardiovascular risk factor; additionally, they found that the detection of EAT attenuation by their DL algorithm demonstrated a significant inverse correlation with the occurrence of cardiovascular events at follow-up.

Finally, two other studies used a combined AI powered radiomics approach to demonstrate the incremental value of assessing PCAT attenuation over traditional CCTA based cardiovascular risk prediction tools (25, 26).

AI-powered detection of imaging biomarkers shows the potential to impact individual cardiovascular risk stratification, a fundamental process to guide the selection of the most appropriate invasive or non-invasive diagnostic testing for CAD patients.

## AI Applications for Cad Diagnosis and Risk Stratification

Functional non-invasive ischemia testing is recommended in patients with high PTP (i.e., >15%) or known CAD; according to the last ESC Guidelines, non-invasive functional imaging should be primarily used to detect ischemia (2).

Myocardial ischemia can be detected through rest or stress induced wall motion abnormalities (RWMA) with stress echocardiograph and areas of reduced myocardial perfusion with stress cardiac magnetic resonance (S-CMR) and with nuclear radiology techniques.

In this section, we will summarize the principal AI applications developed for functional imaging.

## AI Applications to Rest and Stress Echocardiography

Echocardiography is the most available imaging tool for the management of CAD patients. As aforementioned, stress echocardiography is recommended as one of the functional non-invasive imaging tests of choice for the detection of new onset coronary artery disease in the follow-up of CCS patients (2).

The impact of AI applications in echocardiography has been steadily growing: first applications served to improve image quality; gradually, the focus shifted to automatic diagnostic echocardiographic window classification and measures assessment (27).

AI solutions have mainly focused in reducing the high inter-observer variability in the evaluation of regional wall motion abnormalities (RWMA) with rest and stress echocardiography (Table 2).

**Table 2:** Main AI applications to rest and stress echocardiography.

**References**	**Summary**	**Performance**
**Rest echocardiography**		
Raghavendra et al. (28)	Automated detection of RWMAs on rest echocardiograms to identify CAD	96% sensibility and specificity in detecting RWMAs
Kusunose et al. (29)	Automated detection of RWMAs on rest echocardiograms to identify CAD	The DL algorithm performed similar to expert cardiologists in RWMAs detection (AUC 0.99 vs. 0.98; *p* = 0.15) and significantly outperformed the ability of resident physicians (AUC 0.99 vs. 0.9; *p* = 0.002
**Stress echocardiography**		
Mansor et al. (30)	Automated detection of RWMAs on rest and stress echocardiograms to identify CAD	80–85% accuracy in classifying RWMAs
Chykeyuk et al. (31)	Automated detection of RWMAs on rest and stress echocardiograms to identify CAD	93% accuracy in classifying RWMAs
Omar et al. (32)	Comparison of ML and DL algorithms for the automated detection of RWMAs on rest and stress echocardiograms to identify CAD	DL application demonstrated the best accuracy in detecting RWMAs (75% accuracy), followed by the RF (72%) and SVM (71%).

*CAD, coronary artery disease; ML, machine learning; DL, deep learning; RF, random forest; RWMAs, regional wall motion abnormalities; SVM, support vector machine*.

The detection of RWMA on rest echocardiograms was initially attempted using ML methods, which demonstrated high levels of accuracy in distinguishing between normal and infarcted echocardiographic images by correctly identifying the presence of RWMAs (28, 33).

Kusunose et al. (29) demonstrated the application of DL in assessing RWMAs. The authors applied five different DL models to the rest echocardiograms of 300 known CAD patients and 100 age-matched controls. Known CAD patients had an equal distribution of scar myocardium in the territory of left anterior descending, left circumflex and right coronary artery. The five DL models performed similarly to expert cardiologists in detecting RWMAs (AUC 0.99 vs. 0.98; *p* = 0.15) and significantly outperformed the ability of resident physicians (AUC 0.99 vs. 0.9; *p* = 0.002).

Initial attempts to apply AI to stress echocardiography were made using techniques of supervised ML. The first examples date back to 2008 and 2011, when Mansor et al. (30) used a Hidden Markov Model (HMM) to develop a cardiac wall segment model for a normal and an abnormal heart and tested it on rest, stress and combined rest and stress sequences in a relatively small dataset of 44 dobutamine stress echocardiograms (DSE), reaching an accuracy in classifying RWMA of 80–85% with the analysis of combined rest and stress sequences. Few years later, Chykeyuk et al. (31) improved this result using a Relevance Vector Model in a dataset of 173 DSE reaching an accuracy of 93%.

Omar et al. (32) compared different ML and DL algorithms in detecting RWMAs at stress echocardiography. A DL application using a CNN demonstrated the best accuracy by achieving a 75% accuracy, followed by the RF (72%) and SVM (71%).

AI applications (both with ML and DL) to rest and stress echocardiography have shown good results in detecting RWMAs for CAD diagnosis. In particular, they demonstrated high accuracy both with rest and stress images, improving on the high inter-observer variability experienced in human evaluation. Further developments and test cohorts are required for the wide-spread clinical implementation of AI in real-life echocardiographic workflow for CAD diagnosis.

## AI Applications to Stress Cardiac Magnetic Resonance

S-CMR is a powerful diagnostic tool that allows a comprehensive evaluation of known or suspected CAD patients.

Different from other techniques, S-CMR combines the evaluation of global cardiac function with an accurate and reproducible definition of regional myocardial viability by combining information on cardiac muscle function, tissue characterization, persistent and inducible ischemia (34).

Below, we will summarize AI applications to S-CMR for the assessment of cardiac function, tissue characterization and rest and stress myocardial perfusion (Table 3).

**Table 3:** Main AI applications to stress cardiac magnetic resonance (S-CMR).

**References**	**Summary**	**Performance**
**Assessment of cardiac function**		
Bai et al. (35)	Automated myocardial segmentation using a DL algorithm trained in a huge dataset (>4,500 subjects)	Excellent correlation with manual measurement (Dice's coefficient 0.94 for the LV cavity, 0.88 for the LV myocardium and 0.90 for the RV cavity)
Curiale et al. (36)	Automated LV quantification using DL	Good accuracy for myocardial segmentation (Dice's coefficient 0.9); high correlation index for LVEDV and LVESV (0.99), LV EF (0.95), and for SV and CO (0.93).
**Tissue characterization**		
Kotu et al. (37)	Arrhythmic risk stratification of CAD patients through the radiomic analysis of the scar tissue	Highly accurate (94%) classification of CAD patients in high- and low arrhythmic risk groups
Xu et al. (38)	Automated detection of MI	94% overall accuracy in detecting the MI area extension, position and shape
Larroza et al. (39)	Distinction of acute and chronic MI on CMR-LGE and non-enhanced CMR through an ML model combined with radiomics	High AUC, sensitivity and specificity in the distinction between acute and chronic MI both on CMR-LGE (0.86, 0.81, and 0.84, respectively) and on non-enhanced CMR (0.82, 0.79, and 0.80, respectively)
Larroza et al. (40)	Automated identification of myocardial transmural scar on non-enhanced CMR	Sensitivity of 92% for transmural scar identification
Baessler et al. (41)	Automated scar detection on non-enhanced CMR images with a combined ML and radiomics algorithm	Identification of five independent texture features, which allowed scar identification. The best features combination allowed an AUC of 0.93 and 0.92 for diagnosing large and small MI, respectively
Moccia et al. (42)	Comparison of two DL scar segmentation protocols for automated scar detection on CMR-LGE images	88% median sensitivity and 71% DICE similarity coefficient by the protocol that limited the analysis to the myocardial region.
Zabihollahy et al. (43)	Semiautomated DL method for LV myocardial scar segmentation from 3D CMR-LGE images.	94% DICE similarity coefficient for LV myocardial scar segmentation
Zhang et al. (44)	Automated detection, localization and quantification of myocardial fibrosis on non-enhanced CMR	No difference between non-enhanced cardiac cine and CMR-LGE analyses: number of scar segments (*p* = 0.38), mean per-patient scar area (*p* = 0.27) percentage of damaged myocardial tissue (*p* = 0.17)
Ma et al. (45)	Combination of radiomics and T1 mapping for the automated identification of MVO	Radiomics combined with T1 values compared to T1 values alone better identified MVO (AUC 0.86) and showed higher predictive value for LV longitudinal systolic myocardial contractility recovery (AUC 0.77).
**Perfusion S-CMR**		
Scannell et al. (46)	Automated processing and segmentation myocardial perfusion data on S-CMR	High accuracy compared to manual processing and segmentation (Dice similarity coefficient for myocardial segmentation 0.8)
Xue et al. (47)	Automated assessment of MBF on S-CMR	High accuracy compared to manual analysis in myocardial segmentation (Dice similarity coefficient 0.93). No difference in the per-sector MBF identification (*p* = 0.92)

*CAD, coronary artery disease; CO, cardiac output; EF, ejection fraction; LGE, late-gadolinium enhancement; LV, left ventricle; LVEDV, left ventricle end diastolic volume; LVESV, left ventricle end systolic volume; MBF, myocardial blood flow; MI, myocardial infarction; MVO, microvascular obstruction; SV, stroke volume; SVM, support vector machine*.

### Cardiac Function

The first AI applications to S-CMR were focused on semi-automated myocardial segmentation on cine CMR images, in order to speed-up the manual time-consuming process of endo-and epicardial border definition. Many applications have sought to automate the analysis of cine CMR images (36, 48).

Although highly accurate, the majority of these applications were tested on small training datasets, thus limiting their real-life applicability. Bai et al. (35) overcame this limitation by applying a DL algorithm for myocardial segmentation in the UK Biobank, thus training the CNN on the cine CMR images of more than 4,500 patients. When applied on a test set of 600 patients, the DL application showed excellent correlation with manual measurements, with a mean absolute difference of ~6 mL for left ventricular end-diastolic volume (LVEDV), 5 mL for left ventricular end-systolic volume (LVESV) and 7 g for left ventricular mass.

### Tissue Characterization

The correct identification and quantification of the areas of late gadolinium enhancement (LGE) on CMR images portends a well-established prognostic role in CAD patients (49).

**Figure 3:**
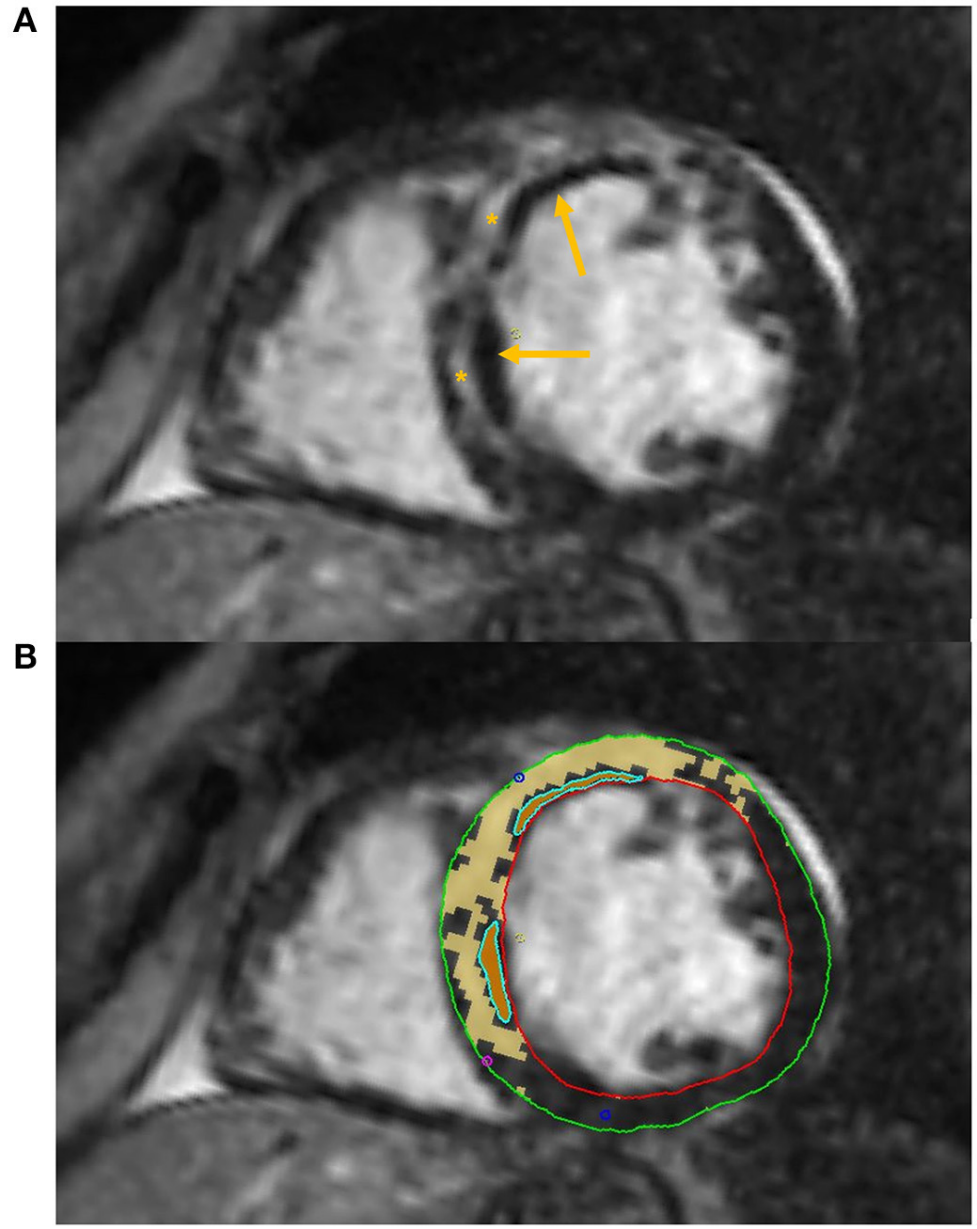
Tissue characterization with Cardiac Magnetic Resonance (CMR). The figure shows an example of tissue characterization in a patient with a history of antero-septal myocardial infarction. **(A)** Depicts a transmural myocardial scar (asterisks) in the antero-septal region with concomitant evidence of subendocardial areas of microvascular obstruction (MVO, arrows). **(B)** Shows the automatic identification and quantification of late gadolinium enhancement and the semi-automatic identification of MVO areas on CMR images.

Some authors have successfully applied DL algorithms to perform automated LGE quantification (Figure 3).

Xu et al. (38) proposed an end-to-end DL algorithm composed of three function layers capable of detecting the MI area at the pixel level, thus automatically obtaining the extension, position, and shape of the MI area for each of the 114 patients analyzed, with a classification accuracy of 94%.

Two other authors developed different DL algorithms, both obtaining high DICE similarity coefficients in LGE quantification when compared to manual segmentation. In the first case, Moccia et al. (42) successfully modified and trained an existing DL application based on two CNN (ENet) to segment scar tissue on enhanced CMR images of 30 patients with known CAD. In the second case, Zabihollahy et al. (43), demonstrated an accurate three-dimensional segmentation of myocardial fibrotic tissue by using a semiautomated method using a 3D CNN.

Albeit promising, these studies are still based on small cohorts, thus limiting the applicability in the routine S-CMR workflow.

Zhang et al. (44) successfully developed a DL model capable to detect, localize and quantify myocardial areas of fibrosis in non-enhanced cine CMR of 212 CAD patients and 87 healthy controls. Notably, the authors did not find any difference between non-enhanced cardiac cine and LGE CMR analyses in the number of scar segments (*p* = 0.38), in the mean per-patient scar area (*p* = 0.27), and in the percentage of damaged myocardial tissue (*p* = 0.17). If confirmed on larger sample sizes, the ability of an AI model to correctly quantify myocardial LGE on unenhanced cine CMR images paves the way to the possibility to perform tissue characterization in patients with end stage renal disease on dialysis, which represents a contraindication to perform contrast enhanced CMR.

Other authors developed AI applications capable of assessing myocardial viability and scar area without the use of LGE with the combination of traditional AI models and radiomics. In two consecutive studies Larroza et al. (39) demonstrated the possibility to use a support vector machine (SVM) combined with radiomics texture analysis to distinguish between acute and chronic MI with similar sensitivity and specificity between analyses conducted on enhanced and non-enhanced cine CMR images and to identify non-viable myocardial segments (i.e., segments with LGE ≥ 50% transmural extension) in non-enhanced cine MRI sequences with a sensitivity of 92% (40).

Similarly, Baessler et al. (41) used a ML algorithm to select five independent texture analysis features to differentiate between ischemic scar and normal myocardium on non-enhanced cine MR images of 120 patients with chronic or subacute MI.

A recent study by Ma et al. (45) has also proven the ability of texture analysis combined with native T1 mapping values to better identify microvascular obstruction (MVO, Figure 3) compared to T1 mapping alone in a small group of patients with recent ST-segment-elevation MI. Combined radiomics features and native T1 values also provided a higher predictive value for LV longitudinal systolic myocardial contractility recovery compared to T1 values in a subset of patients that underwent 6-months follow up CMR.

Finally, Kotu et al. (37) provided an interesting proof of concept of how radiomics can help in CAD risk stratification. The authors successfully created a radiomic algorithm able to perform a correct risk-stratification for the occurrence of life-threatening arrhythmias in 34 known CAD patients on the basis of the radiomic analysis of the scar tissue.

Albeit deeply interesting, radiomics studies applied to CMR are still based on small datasets and currently does not appear feasible for large-scale routine use. Further investigations on larger CMR datasets are needed to broaden the spectrum of use.

### Perfusion S-CMR

S-CMR can detect hemodynamically significant CAD through the assessment of myocardial ischemia through the evaluation of perfusion defects. In routine clinical practice, the analysis of S-CMR images is performed qualitatively, through the visual assessment of S-CMR images by an expert reporting physician.

First pass gadolinium enhanced CMR perfusion imaging has shown the potential to delineate a fully quantitative assessment of myocardial blood flow (MBF). Fully automatic MBF maps have been validated against gold standard perfusion techniques, such as positron emission tomography (50).

Albeit highly accurate in CAD diagnosis (51), quantitative S-CMR perfusion is time consuming and therefore restricted to research purposes (52).

In recent years, innovative AI applications have been developed to allow fully automated perfusion mapping approaches to enter clinical practice.

Preliminary work by Scannell et al. (46) successfully developed a DL algorithm to fully automatize image processing for myocardial perfusion assessment.

More recently, Xue et al. (47) validated a CNN model on more than 1,800 CMR rest and stress scans from 1,034 patients. The DL model showed excellent mean Dice similarity coefficient ratio of automatic and manual myocardial segmentation (0.93 ± 0.04) and did not differ significantly from per-sector MBF manual assessment (*p* = 0.92). The same group of authors demonstrated (53) that MBF and myocardial perfusion reserve (MPR, i.e., ratio of stress to rest MBF) automatically assessed using their DL model were independently associated with death and MACE in a cohort of >1,000 patients.

This large, multicenter study paves the way for automatic assessment of MBF and MPR from quantitative CMR perfusion mapping to enter the routine diagnostic workflow of patients undergoing S-CMR.

## AI Applications to Nuclear Imaging for the Detection of Ischemia

Nuclear radiology has been one of the first imaging methods applied to ischemia assessment in CAD patients and still represents the most widely used test to detect myocardial ischemia.

Single-photon emission computed tomography (SPECT) and positron emission tomography (PET) represent the two main tools of nuclear imaging applied to cardiology.

Despite its relative low cost and discrete accuracy in detecting CAD, the detection of ischemia by SPECT analysis mostly relies on qualitative methods and appears prone to possible CAD underestimation, especially in patients with non-obstructive multivessel coronary artery disease. PET is able to provide robust quantitative analysis of myocardial blood flow and can detect microvascular ischemia. However, the utilization of PET analysis is limited by its high technical complexity and high costs (54).

Apart from those aimed at image pre-processing and segmentation (55), the major AI applications to myocardial perfusion SPECT focused on boosting the power of cardiac nuclear imaging in two principal tasks: to identify patients with obstructive CAD and to define their prognosis (Table 4).

**Table 4:** Main AI applications to nuclear imaging for the detection of ischemia.

**References**	**Summary**	**Performance**
**Identification of patients with obstructive CAD**		
Arsanjani et al. (56)	Comparison of automated quantification of myocardial perfusion SPECT to expert visual analysis	AUC for TPD was significantly better compared to visual evaluation of two expert analysis (0.91 vs. 0.87 and 0.89, *P* < 0.01).
Arsanjani et al. (57)	Comparison of automated quantification of myocardial perfusion SPECT integrated with clinical information to expert visual analysis and traditional TPD quantification	ML diagnostic accuracy (87%) was similar to Expert 1 (86%), but superior to TPD quantification (83%) and Expert 2 (82%) (*P* < 0.01).
Betancur et al. (58)	Automated prediction of obstructive CAD by DL algorithm on SPECT as compared with total perfusion deficit (TPD)	DL AUC for disease prediction was higher than for TPD (per patient analysis: 0.80 vs. 0.78; per vessel analysis: 0.76 vs. 0.73: *p* < 0.01)
Otaki et al. (59)	Automated prediction of obstructive CAD by externally validated DL algorithm on SPECT as compared to expert visual analysis and with total perfusion deficit (TPD)	DL AUC for obstructive CAD detection was higher than for TPD and visual assessment (0.80 vs. 0.73 and 0.65, respectively). The algorithm was self-explainable and externally validated
**Prognostic evaluation**		
Arsanjani et al. (60)	Application of ML algorithm to SPECT analysis to predict early revascularization in patients with suspected CAD	The ML algorithm showed similar sensitivity for prediction of revascularization to expert visual assessment (74% for both) with a better specificity he specificity of ML (75 vs. 67%, *P* < 0.05)
Betancur et al. (61)	MACE risk prediction with a ML application integrated with clinical and SPECT imaging features	3-years MACE prediction by ML application combined with clinical data outperformed ML with imaging data alone (AUC: 0.81 vs. 0.78) and showed also higher predictive accuracy compared with expert evaluation and automated TPD (AUC: 0.81 vs. 0.65 vs. 0.73, respectively)
Hu et al. (62)	Efficacy of per-vessel prediction of early revascularization compared among ML application, expert evaluation and standard TPD quantification	The per-vessel and per-patient AUC of early revascularization prediction (0.79 and 0.81, respectively) was higher than by TPD (*p* < 0.001) and outperformed qualitative experts' interpretation

*AUC, area under the curve; CAD, coronary artery disease; SPECT, single positron emission tomography; TPD, total perfusion deficit*.

In 2013, Arsanjani et al. published two different studies on relatively large populations. The first one (56) demonstrating that a fully automated quantification of myocardial perfusion SPECT was equivalent on a per-patient level and superior on a per-vessel level, in detecting significant coronary artery stenosis (i.e., ≥ 70%) when compared with expert visual analysis. The second paper (57) analyzed the application of a ML LogitBoost model which integrated quantitative perfusion and clinical data to a dataset of 1,181 myocardial perfusion SPECTs. The AI application significantly outperformed the visual qualitative analysis of two expert readers who were provided with the same imaging, quantitative, and clinical data.

More recently, Betancur et al. (58) introduced the possibility to use a DL algorithm for the analysis of myocardial perfusion SPECT. The authors trained their application on a large dataset of more than 1,500 myocardial perfusion SPECT polar maps. As previously demonstrated for the ML applications proposed by Arsanjani et al., the DL algorithm improved the identification of patients with obstructive CAD, compared to standard clinical evaluation.

Finally, Otaki et al. (59) recently introduced a novel DL algorithm for the detection of obstructive CAD following SPECT myocardial perfusion imaging. The AI application was first developed in a dataset of more than 2,000 patients and then externally tested in 555 patients with excellent AUC compared to traditional TPD quantification and expert visual assessment (AUC 0.80, 0.73, and 0.65, respectively). External validation of AI applications represents a fundamental step to obtain the fast implementation of AI algorithms in clinical practice and will soon be required for every newly developed AI algorithm.

ML and DL models have also been applied to myocardial perfusion SPECT to improve its prognostic value.

Arsanjani et al. (60) demonstrated how a ML application could improve the prediction of early revascularization in patients with suspected CAD undergoing perfusion SPECT. The authors developed a ML LogitBoost model that integrated clinical data and quantitative features derived from perfusion SPECT. When tested on 713 rest perfusion SPECT scans, the ML application showed comparable or better performance with respect to expert readers in predicting early revascularization.

Recently, Hu et al. (62) provided a more robust example of a ML algorithm able to perform a per-vessel prediction of early coronary revascularization (i.e., within 90 days) after SPECT myocardial perfusion imaging. To do so, the authors developed and tested ~2,000 patients using a ML algorithm that integrated multiple clinical, stress test and SPECT imaging variables and compared its performance with standard quantitative SPECT analysis (i.e., total perfusion deficit, TPD) and expert evaluation. The LogitBoost application outperformed automatic myocardial perfusion quantitation by TPD and expert's interpretation.

Finally, Betancur et al. (61) developed a robust ML application integrated with clinical and imaging features. This model demonstrated high predictive accuracy to determine the risk of major cardiovascular adverse events (MACE) in a large population of 2,619 patients followed for ~3 years. The algorithm demonstrated its superiority over all existing visual or automated perfusion assessments.

## AI Applications for Coronary Computed Tomography Angiography

Initial applications of CCTA in CAD management were on the anatomical detection or exclusion of obstructive CAD, with CCTA progressively assuming the role of gatekeeper to unnecessary ICAs. Due to its high negative predictive power, CCTA has been indicated as the preferred test to rule out CAD in low to intermediate clinical PTP patients by the most recent ESC Guidelines on the management of CCS (2).

However, CCTA has rapidly advanced beyond the qualitative anatomical assessment of the presence of obstructive CAD and is now capable of offering a complete anatomical and functional characterization of CAD, thus providing important diagnostic and prognostic (63) information for patients' management.

In this section, we will review the principal AI applications developed for the anatomical and functional assessment of CAD with CCTA (Table 5).

**Table 5:** Main AI applications to Coronary Computed Tomography Angiography (CCTA).

**References**	**Summary**	**Performance**
**Coronary stenoses grading**		
Kelm et al. (64)	Automated ML detection, grading and stenoses grading on CCTA images	Good sensitivity and specificity (95 and 67%) compared to expert evaluation to correctly detect significant coronary artery stenoses
Kang et al. (65)	Automated ML detection of coronary artery stenoses on CCTA images	High sensitivity (93%), specificity (95%), and accuracy (94%), with AUC (0.94) for coronary artery stenoses detection compared to experts' visual assessment
Zreik et al. (66)	Automated LV myocardium analysis to identify patients with significant coronary artery stenoses	The DL application correctly performed LV segmentation (Dice similarity coefficient 0.91) and identified patients with significant coronary artery stenosis with an AUC value of 0.74
Hong et al. (67)	Automated DL coronary artery stenoses grading (plaque segmentation, MLA and percent DS quantification) on CCTA images	Excellent correlation of ML performance to expert readers (ρ = 0.984 for MLA; ρ = 0.957 for DS *p* < 0.001 for all)
Muscogiuri et al. (68)	Automated DL classification of coronary artery stenoses according to CAD-RADS	The DL algorithm showed its best performance in differentiating between CADRADS 0 (i.e., no coronary atherosclerosis) vs. CADRADS > 0 (i.e., detectable coronary atherosclerosis) with a sensitivity of 66% and a specificity of 91%, compared to experts' analysis
**Plaque phenotype characterization**		
Dey et al. (69)	Automated distinction between calcified and non-calcified plaques	Strong correlation between automated plaque analysis and expert readers (ρ = 0.94, for NCP volume; ρ = 0.88, for CP volume; ρ = 0.90 for NCP and CP composition)
Kolossváry et al. (70)	Identification of radiomic features associated to the presence of NRS in coronary artery plaques	Identification of NRS through radiomic analysis with an AUC > 0.92. One radiomic feature reached a remarkable AUC of 0.92 for NRS identification
Masuda et al. (71)	Automated ML algorithm for the detection of fibrous or fibro-fatty coronary artery plaques	The ML algorithm identified high risk coronary plaques better than intravascular ultrasound evaluation (AUC 0.92 vs. 0.83)
Zreik et al. (72)	DL application to perform a complete anatomical coronary artery assessment (stenosis grading associated to plaque features analysis)	Good accuracy in plaque phenotype characterization (AUC 0.77) and in determining its anatomical significance (i.e., stenosis degree above or below 50%, AUC 0.80)
Han et al. (73)	Automated ML algorithm to identify RPP	The ML model that included clinical variables, qualitative and most importantly quantitative plaque features showed the highest performance in identifying patients at risk of RPP (AUC 0.83)
Choi et al. (74)	DL application to perform a complete anatomical coronary artery assessment (stenosis grading associated to plaque features analysis) and CAD-RADS classification	Accuracy compared to three expert readers' analysis for stenoses >70%: 99.7%; accuracy for stenoses>50%: 94.8%. Excellent concordance in CAD-RADS classification with expert readers: agreement within one CAD-RADS category: 98% exams per-patient; 99.9% vessels on a per-vessel basis.
**AI powered CT-FFR**		
Coenen et al. (75)	Definition of the diagnostic accuracy of a ML application to CT-FFR	In the per-vessel analysis, ML-CT-FFR improved diagnostic accuracy by 20% compared to CTA (from 58 to 78%). The per-patient accuracy improved by 14% compared to CTA (from 71 to 85%). Seventy-three percent false-positive CTA results were correctly reclassified by ML-CT-FFR
Nous et al. (76)	Feasibility of ML-CT-FFR application in patients with DM	Overall diagnostic accuracy of ML-CT-FFR in diabetic patients was higher (83%) than in non-diabetic patients (75%); AUC 0.88 and 0.82 for diabetic and non-diabetic patients, respectively
Baumann et al. (77)	Differences in ML-CT-FFR application between patients of different genders	ML-FFR-CT equally performed in both genders, not showing significative difference in the AUC between males (0.83) and females (0.83)
Tesche et al. (78)	Feasibility of ML-CT-FFR application in the presence of heavy calcifications	No statistically significant differences in the diagnostic accuracy, sensitivity, or specificity of ML-CT-FFR were observed across CT scans of patients attributed to different Agatston score categories

*CAD-RADS, Coronary Artery Disease Reporting and Data System; DM, diabetes mellitus; CP, calcified plaque; DS, diameter stenosis; FFR, fractional flow reserve; MLA, minimal luminal area; NCP, non-calcified plaque; NRS, Napkin ring sign; RPP, rapid plaque progression*.

### Coronary Stenoses Grading

The degree of luminal stenosis and the localization of CAD with CCTA has a defined prognostic role (63). AI applications are trying to automate and standardize the process of coronary image reconstruction, segmentation and stenosis degree quantification, which currently relies on visual assessment and is troubled by high inter operator variability (79). Recently AI applications have also focused to significantly shorten reporting time (80), also with non-optimal images, i.e., in the presence of heavy calcifications or in the case of scarce image quality (81).

In 2011, Kelm et al. (64) developed one of the first examples of an AI algorithm capable of correctly analyzing CCTA images to detect, grade and classify as significant coronary stenoses caused by all types of plaques. The ML algorithm was composed of a multistep approach that included automatic centerline verification and lumen cross section estimation and showed good values of sensitivity and specificity (i.e., 95 and 67%, respectively) when compared with expert qualitative evaluation. Importantly, the time required for the ML algorithm to analyze each case was only 1.8 s.

Later, Kang et al. (65) used a different ML algorithm, which showed an even improved accuracy in CAD detection, despite a further reduction in the time required for analysis (only 1 s per case).

In 2019, Hong et al. (67), validated a DL algorithm with CNN across a dataset of 156 CCTAs. The application automatically performed coronary lumen and plaque segmentation and computed minimal luminal area (MLA), percent diameter stenosis (DS) and percent contrast density difference (CDD) with excellent correlation to expert readers (*r* = 0.984 for MLA; *r* = 0.957 for DS; and *r* = 0.975 for CDD, *p* < 0.001 for all).

**Figure 4:**
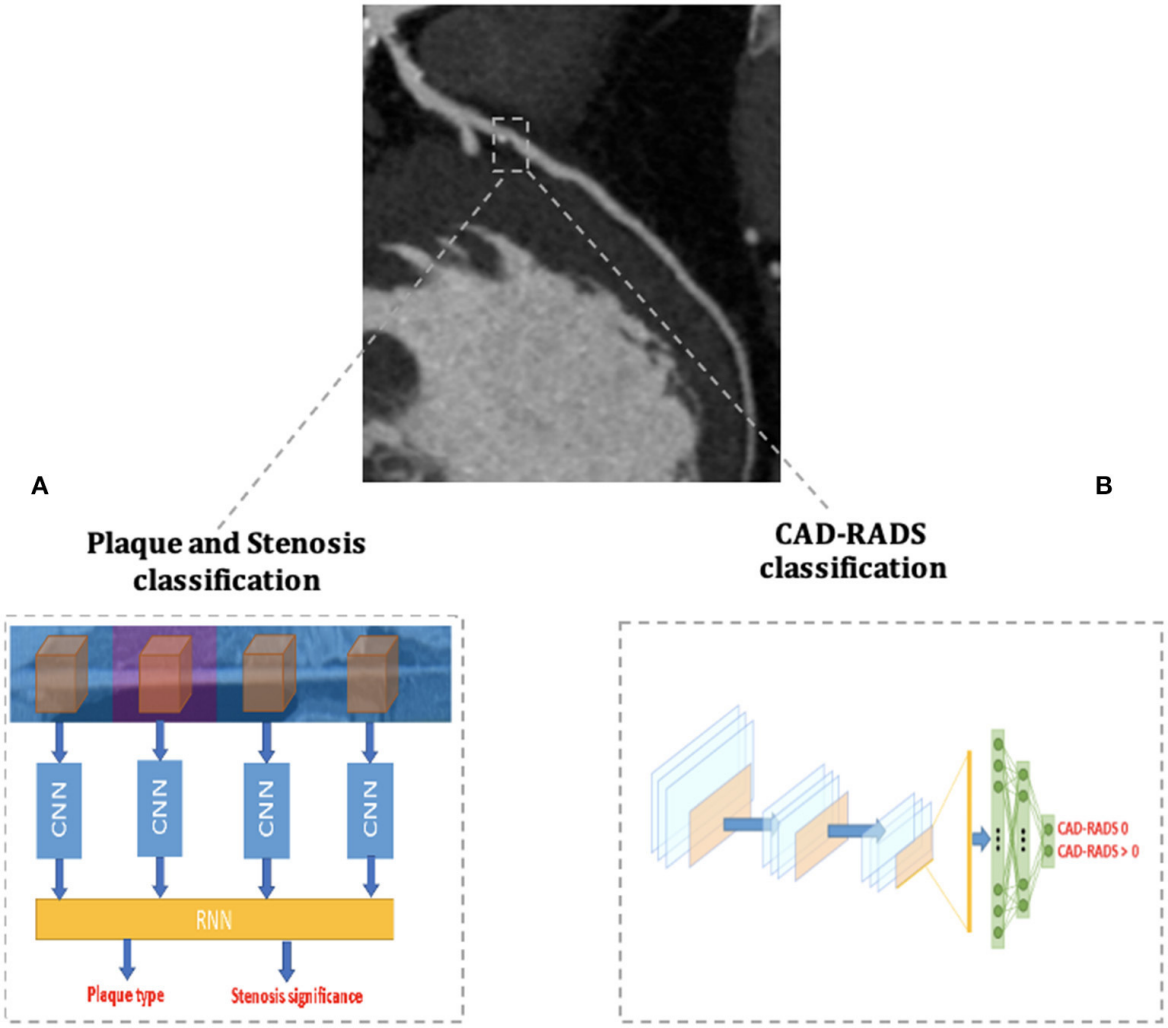
AI applications to cardiac CT. The figure depicts two examples of AI workflow applied to the automatic analysis of coronary artery stenoses by Coronary Computed Tomography Angiography (CCTA). **(A)** Schematizes the algorithm proposed by Zreik et al. (72) and composed of a 3D convolutional neural network (CNN) used for coronary artery features extraction and a subsequent recurrent neural network for a two-task classification of plaque phenotype and stenosis degree. **(B)** Schematizes the algorithm applied by Muscogiuri et al. (68) for the fully automatic CAD-RADS classification of CCTA scans with a CNN.

Recently, Muscogiuri, et al. (68) demonstrated good results of a DL CNN in classifying CCTAs examinations in the correct category of an existing reporting system (namely the Coronary Artery Disease Reporting and Data System, CAD-RADS) (Figure 4). If confirmed on larger datasets, this study paves the way to the use of a CNN algorithm in clinical practice to rule out the presence of CAD in a relatively short time, reducing referring physicians' workload and helping them in focusing only on pathological CCTAs.

An alternative approach for assessing the presence of hemodynamically significant coronary artery stenosis is the one proposed by Zreik et al. (66), who demonstrated that a DL algorithm could perform an automatic analysis of the LV myocardium in a single CCTA scan acquired at rest, without assessment of the anatomy of the coronary arteries, to identify patients with functionally significant coronary artery stenosis with an AUC value of 0.74. When applied to a dataset of 100 CCTAs with intermediate grade coronary artery stenosis (82), the implementation of this DL application to the quantification of stenosis degree outperformed the traditional method of anatomical stenosis evaluation alone (AUC 0.76 and 0.68, respectively).

### Plaque Phenotype Characterization

One of the key advantages of CCTA for the assessment of CAD is the ability to fully characterize coronary plaque phenotype.

**Figure 5:**
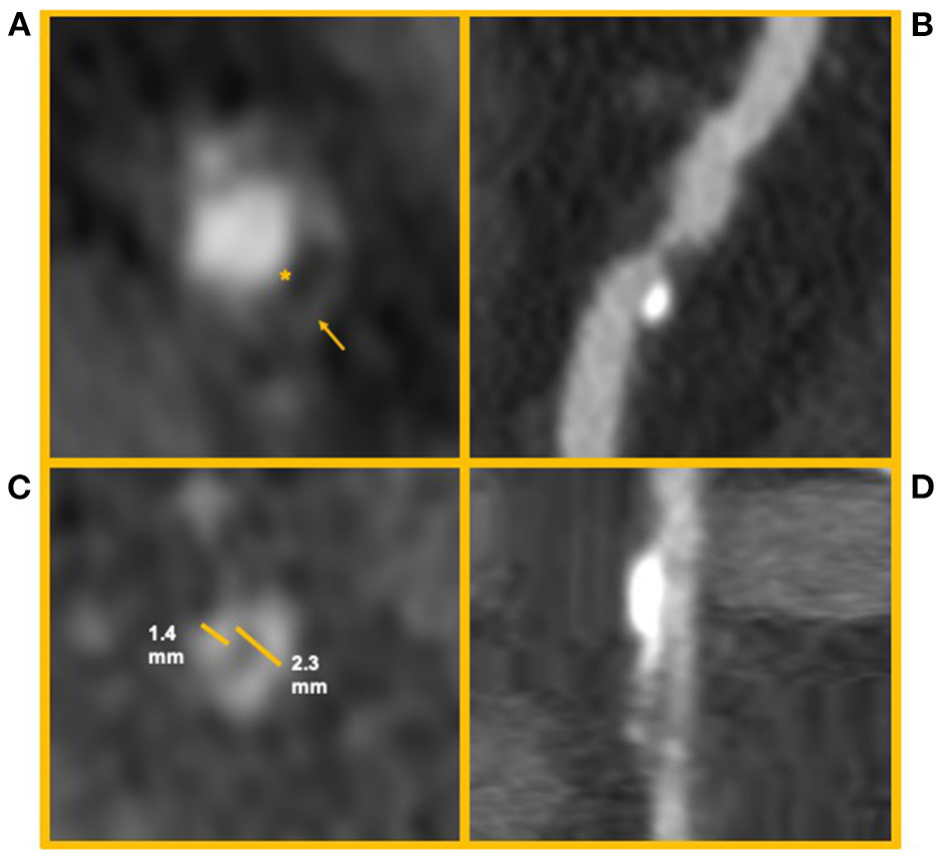
High risk features of coronary artery plaques. The figure shows clinical examples of high-risk plaque feature. **(A)** Depicts an example of a Napkin-ring sign. The asterisk identifies the hypodense necrotic core; the arrow identifies the hyperdense ring-like thin cap. **(B)** Depicts a high-risk coronary plaque with spotty calcifications and low attenuation (i.e., attenuation <30 HU). **(C,D)** Depict a coronary artery plaque with positive remodeling (i.e., a positive ratio between diameter of the vessel outside the plaque and its internal diameter) in short and long axis, respectively.

Not all coronary lesions imply the same cardiovascular risk. In particular, the detection of prevalent fibrotic composition and other specific plaque features at CCTA (Figure 5) have been associated with an increased risk of cardiovascular events (83). These high-risk features are represented by spotty calcifications, positive remodeling, low attenuation, and the napkin-ring sign (NRS) (83).

AI applications have been developed to automate this process, in order to provide the clinician a full set of information to guide patient management.

One of the first AI approaches demonstrated the ability of an automated algorithm to correctly classify calcified and non-calcified lesions compared to expert manual quantification (69).

Masuda et al. (71) applied a ML histogram algorithm for the automatic detection of fibrous or fibro-fatty coronary plaques with CCTA. The ML method significantly outperformed the conventional CT parameters in the identification of high-risk plaques, when compared to intravascular ultrasound (IVUS) evaluation (AUC 0.92 vs. 0.83, respectively; *p* = 0.001).

Another feature correlated with high risk of cardiovascular events is rapid plaque progression (RPP), defined as an annual progression of percentage atheroma volume ≥1.0%. The study by Han et al. (73) provided an interesting demonstration of how a ML framework that incorporated clinical information together with qualitative and quantitative CCTA plaque parameters could better discriminate at risk patients compared to traditional risk scores and also ML models that incorporated only clinical or clinical and qualitative variables together.

This and other ML integrated clinical and CCTA parameter risk scores have shown the potential to clearly outperform traditional CCTA risk score evaluations (73, 84) when applied in large cohorts with long follow-up.

In the future, the application of these AI empowered risk scores will enhance risk prediction in CAD patients, ultimately boosting the power of CCTA and other imaging exams.

Zreik et al. (72) developed a comprehensive anatomical DL application trained to analyze both the presence of significant CAD (i.e., the presence of stenoses with ≥50% of luminal narrowing) and to classify the phenotype of coronary artery plaques. To do so, the DL application was structured with a 3D CNN used to extract features of each coronary artery and with a recurrent neural network used to perform the two simultaneous classification tasks (Figure 4). The algorithm was validated on CCTA scans of 163 patients and reached good levels of accuracy in plaque phenotype characterization (0.77) and in determining its anatomical significance (0.80). If validated on larger cohorts, this comprehensive approach allows the clinician to maximize the anatomical evaluation of coronary artery plaques with CCTA.

Recently also Choi et al. (74) (CIT) proposed a new AI application capable of performing a comprehensive anatomical plaque quantification with impressive value of accuracy when compared to the analysis of three expert readers (accuracy for stenoses >70%: 99.7%; accuracy for stenoses>50%: 94.8%). Notably, the algorithm was also able to classify patients according to the CAD-RADS score with an excellent concordance with expert readers agreement within one CAD-RADS category in 98% exams per-patient and 99.9% vessels on a per-vessel basis.

Another innovative approach to coronary plaque characterization has been represented by the combination of radiomics with AI applications, which has shown the potential to provide useful information on high-risk coronary plaque features.

Kolossvary et al. (70) detected more than 400 radiomic features that significantly differed between plaques with and without napkin-ring sign, reaching an AUC > 0.8. Among these, one parameter called “short run low gray-level emphasis” reached an impressive AUC of 0.92 in NRS plaque identification.

The same authors expanded the previous observation by demonstrating that CCTA radiomics could identify invasive and radionuclide imaging markers of plaque vulnerability significantly better than traditional quantitative and qualitative CT parameters (85).

## AI for Functional Ischemia Assessment by Cardiac CT

### AI Powered CT-FFR

Traditional CCTA techniques only provide anatomical assessment of CAD. In cases of coronary atherosclerosis of uncertain hemodynamic significance, current guidelines support the use of an ischemia test to assess the need for revascularization (2).

In recent years, the development of a non-invasive method to calculate CT-derived fractional flow reserve has permitted the evaluation of the anatomical and functional hemodynamic significance of coronary artery lesions (86–90). This approach has been validated in numerous studies (86, 91) against gold standard invasive FFR and resulted in high diagnostic accuracy in detecting hemodynamically significant stenosis and in determining their prognostic impact (92), especially when combined with information regarding coronary plaque phenotypes (54, 93).

Importantly, Rabbat et al. (94) studied 431 patients who underwent a CCTA alone vs. CCTA + FFRCT diagnostic pathway and demonstrated the safe deferral of ICA in patient with stable CAD who underwent the CCTA + FFRCT strategy. FFR_CT_ was feasible with a conclusive result in >90% of patients. Among those who deferred ICA, there were no major adverse cardiac events. A high proportion of those who underwent ICA were revascularized, resulting in higher diagnostic ICA yield and more efficient utilization of catheterization lab resources.

Multiple AI applications have been developed in recent years to automate the assessment of CT-FFR (75, 95).

Notably, the application of ML to CT-FFR has been clinically validated in a retrospective trial called MACHINE Registry that involved five different centers in Europe, USA and Asia. The ML based computation of CT-FFR outperformed CCTA in terms of diagnostic accuracy; when compared to invasive FFR, ML CT-FFR showed 78% accuracy in comparison to the 58% accuracy of visual CTA alone and the AUC for the detection of hemodynamically significant coronary artery stenosis favored the ML CT-FFR approach (AUC 0.84 vs. 0.69 for CCTA alone). ML CT-FFR was capable of correctly reclassifying 73% false-positive CTA results.

The ML application to CT-FFR in the MACHINE registry were later confirmed in multiple sub-studies that investigated their reproducibility in different clinical scenarios: in particular, ML CT-FFR proved its feasibility in the presence of heavy coronary calcifications (78); in patients with diabetes mellitus (76) and between genders (77).

Poor image quality and high heart rate represent potential limitations to CT-FFR (96).

### CT Perfusion Analysis

The study of myocardial perfusion through cardiac CT is a powerful and promising tool, since it can provide combined anatomical and functional information for every coronary territory with a single test. CT perfusion (CTP) is able to detect obstructive CAD better than CCTA alone (97) and is non-inferior to CMR in the functional evaluation of hemodynamically significant coronary artery stenosis (98).

Despite the great potential of this technique, to date only a few examples of AI applications to CTP exist in the literature and are based on the ML algorithm's ability to assess defects of myocardial perfusion from CTA images acquired at rest (99, 100).

In the future, AI applications have the potential to dramatically impact the field of CTP with applications focused on automatic myocardium segmentation and perfusion defect identification. A particular advantage of this algorithm will be the possibility to directly correlate the presence of anatomically detectable obstructive CAD with the functional evaluation of specific lesions with CTP sequences.

This approach has particular potential in complex CCTAs analysis, for example in the presence of previously revascularized vessels.

## Discussion

The implementation of AI applications to the multimodality imaging applied for the diagnosis and risk stratification of CAD patients represents a new frontier in cardiology. The implementation of AI in the clinical workflow will impact different aspects of the routine clinical workflow.

First, it will offer the possibility to shorten reporting time and to provide pre-reading evaluation of normal exams, to save radiologists and cardiologists time only pathological examinations.

Secondly, it will reduce inter-observer variability in the evaluation of exams. Moreover, it will provide more accurate models of prognostication (7), giving the clinician the possibility to tailor the treatment to the single patient. In this context, unsupervised learning will probably allow to enable the so-called “precision cardiology” by allowing the precision phenotypization of patients, allowing the cardiologist to go beyond the traditional monolithic disease concepts and tailor prognostication and therapy on the single patient features (101).

To spread the application of AI models in real-world clinical practice, however, some potential limitations, pitfalls and ethical considerations need to be considered (102).

As a first limitation, we must acknowledge the vast majority of AI applications have been validated in single-center studies with a limited number of cases and is therefore still restricted to research settings. As aforementioned, in fact, a fundamental principle of AI models is represented by the availability of huge sets of high-quality data for the algorithms to be developed, trained and tested.

In the field of cardiovascular imaging, the availability of large datasets from different centers is particularly crucial to overcome the biases currently present in the development of AI applications.

A first bias is represented by the great variability in terms of exams quality and interpretation (for example in the field of echocardiography) and in the lack in homogeneity in acquisition protocols and machine vendors (for example in the field of cardiac MRI). Therefore, a crucial step to assure AI algorithms generalizability is to perform their development on datasets containing information from machines from multiple vendors and obtained with different acquisition protocols, as in the case of cardiac MRI.

Secondly, in view of the great interobserver variability in some methods such as echocardiography, it is particularly important that the implementation of the training datasets and the quality control process, although time-consuming and costly, is not carried out by a single operator, but by teams of experienced cardiologists, if possible, from different centers. In fact, the risk is that the implementation of quality control by a single operator may lead to the unconscious introduction of new biases into the algorithm, making it usable only within the research group in which it was developed.

Future collaboration among different research groups and hardware vendors will constitute the basis for the development of more generalizable algorithms.

A further possible bias that can limit the general application of AI algorithms is the prevalence of certain ethnic or gender groups within the datasets on which AI applications are developed.

Thinking of a future world in which the use of AI should become routine, it is certainly necessary to ensure that this technology is equally available for all people, independent of gender, social class and ethnic origin. In order to make this possible, several obstacles must be overcome.

First, for AI algorithms to work homogeneously on people of different genders and ethnicities, they would have to be trained on heterogeneous datasets, including different ethnic groups and an equal number of people of both genders. At present, however, women and people from ethnic minorities have been consistently underrepresented in large trial databases (103).

This must be avoided, as it could lead to the exclusion of entire sections of the population from access to the most advanced medical care, thus exacerbating the inequalities already present today. In fact, even though AI applications are in most cases still at an early stage of development, examples have already been reported in the literature of discrimination between different ethnic or economic groups by some AI applications (104).

In order to overcome this possible bias, it is necessary for regulatory agencies to enforce fair inclusion in AI application development databases of patients of different gender and ethnic origin.

Secondly, given the high development costs and the great need of medical records, AI applications will mainly be implemented in high-income countries, based on the organizational needs of their national health systems. This could lead to a substantial inapplicability of AI algorithms in poorer countries, making advanced care even more inaccessible to their citizens.

This could be avoided by devoting some of the public resources allocated to the development of AI algorithms in richer countries to the implementation of applications that improve the quality of care in low-income countries.

Once obtained, robust AI applications will also require validation in large clinical trials to prove benefits in patient care, the economic sustainability and safety of their implementation in routine clinical workflow.

AI application validation in clinical trials will in fact help in overcoming a further issue, which is represented by the medical-legal aspect. In the future doctors will probably base their decisions on risk score algorithms and diagnostic tools powered by AI applications. In this scenario, who will be considered responsible, in the case of a wrong decision made on the basis of incorrect information?

This issue seems even more important if we consider that the majority of AI applications are characterized by a lack of transparency of their intermedium processes: the human operator knows the input data and the result of the elaboration, but can hardly understand the internal algorithm processes.

To overcome this possible pitfall there will be a need to act on two fronts.

On one side, national and transnational medical regulatory authorities will need to further regulate laws and protocols in perspective of a large-scale use of AI algorithms in everyday clinical workflow.

The European Commission on medical AI (105) published a white paper that sought to establish founding principles (such as safety, privacy, data governance transparency, diversity and non-discrimination) for the development of future AI applications, opening up the possibility of supplementing legislation already in place to better protect the health and safety of its citizens.

On the other side, AI developers will need to work on algorithms' self-explicability and internal transparency, to produce so-called explainable AI applications, namely AI software able to provide justification for every stage of their choices, in order to provide the physician all the information needed for thoughtful clinical decision making.

## Conclusions

The medical management of patients with coronary artery disease, one of the most prevalent diseases in the world, is rapidly progressing with the implementation of multimodality imaging in diagnostic and prognostic routine workflows.

AI applications have proven the ability to significantly improve the detection of coronary artery disease with both an anatomical and a functional imaging approach.

Thus, the application of AI to multimodality imaging will continue to play a prominent role in every stage of the diagnostic, risk-stratification and follow-up of patients affected by coronary artery disease. Larger clinical validation and research on safety need to be implemented before large-scale adoption in routine clinical practice.

## Author Contributions

All authors made a substantial contribution to the conception, design of this review paper, including drafting and revisions, and granted their approval for all aspects of the manuscript and its submission.

## Conflict of Interest

The authors declare that the research was conducted in the absence of any commercial or financial relationships that could be construed as a potential conflict of interest.

## Publisher's Note

All claims expressed in this article are solely those of the authors and do not necessarily represent those of their affiliated organizations, or those of the publisher, the editors and the reviewers. Any product that may be evaluated in this article, or claim that may be made by its manufacturer, is not guaranteed or endorsed by the publisher.
